# Nevus-Like Appearance of Primary Malignant Melanoma of the Esophagus

**DOI:** 10.1155/2009/285753

**Published:** 2009-07-27

**Authors:** Min-Jung Kang, Sun Young Yi

**Affiliations:** Department of Internal Medicine, School of Medicine, 158-056 Ewha Womans University, 911-1 Mokdong Yangcheon-Ku, Seoul, South Korea

## Abstract

The primary malignant melanoma of the esophagus (PMME) is a rare malignant disease, accounting for only 0.1–0.2% of all esophageal neoplasms, and the majority of the patients are diagnosed at advanced stages with poor prognosis. We present here a case of 56-year-old woman with epigastric pain and her endoscopic finding revealed several flat and black pigmented mucosal lesions within the distal portion of the esophagus which looked like flat nevus. The histopathology and immunohistochemical profile of the tissue specimens were diagnostic of malignant melanoma.

## 1. Introduction

The primary malignant melanoma of esophagus (PMME) is an extremely rare and highly aggressive tumor with the mean survival time of 10 months and the 5-year survival rate of only 4% [1]. Since the first case of PMME was reported by Baur in 1906, only 262 cases have been documented by June 2005 worldwide [2]. The incidence is from 0.1% ~ 0.2% of all esophageal malignancies [3]. Gross appearances of the PMME, which often locates intraluminally at distal part of the esophagus, are typically solitary, polypoid, and irregularly pigmented [4]. We present here a case of unique endoscopic appearance PMME which looks like flat nevus.

## 2. Case Report

**Figure 1:**
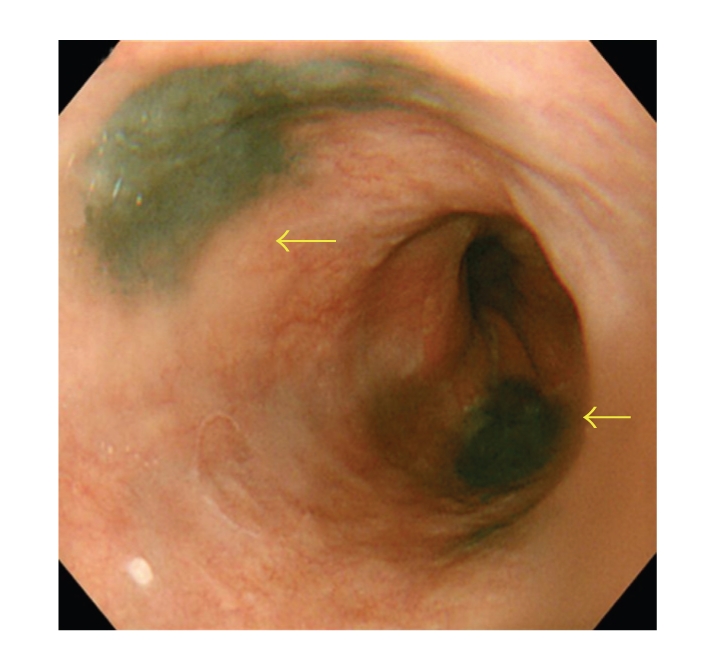
Esophagoscopy showing several flat and black pigmented mucosal lesions in the distal esophagus and esophagogastric junction (arrows).

**Figure 2:**
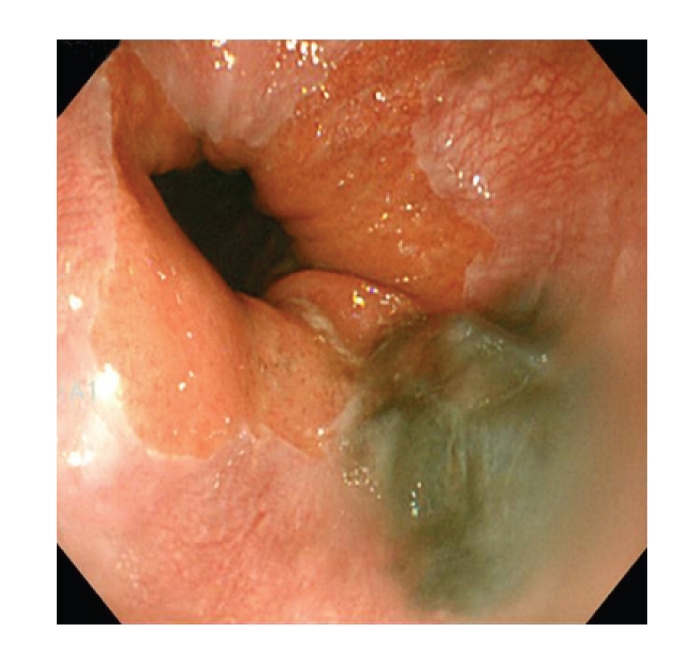
Esophagoscopy showing flat and black pigmented mucosal lesions with reflux esophagitis in close up of esophagogastric junction.

**Figure 3:**
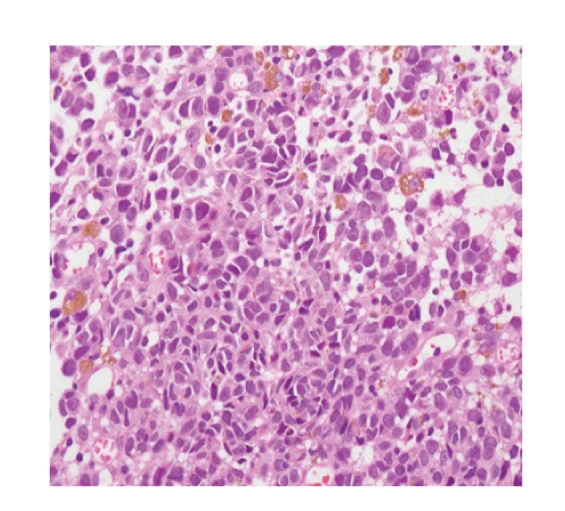
Histologically the tumor mainly consisted of poorly cohesive neoplastic cells with hyperchromic muclei and cytosolic melanin granules which predominantly proliferated in the mucosa (Hematoxylin and eosin stain, ×400).

**Figure 4:**
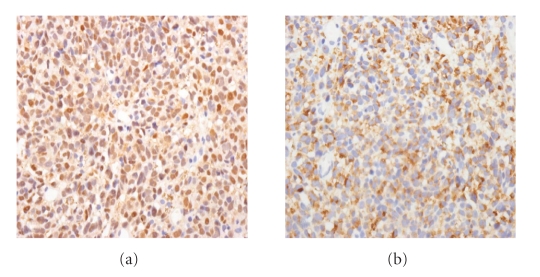
Immunohistochemical stain. The cells sporadically showed immunoreactivity for S-100 protein (×400, a) and HMB-45 antibody (×400, b).

A 56-year-old man presented to our hospital complaining of epigastric pain for 10 days. He had been medically treated for reflux esophagitis, and the gastric ulcers infected secondary to *helicobacter pylori* since 2002. He took a medication of proton pump inhibitor. His physical examination was unremarkable and revealed no evidence of organomegaly or lymphadenopathy. He did not have any other comorbidities. Esophagoscopy revealed several flat and black pigmented mucosal lesions with a short shallow mucosal break in the distal esophagus and esophagogastric junction (Figures 1  and 2). Microscopic examination of biopsy specimens showed proliferation of poorly cohesive neoplastic cells with hyperchromic nuclei and cytosolic melanin granules which predominantly proliferated in the mucosa (Figure 3, H&E stain, ×400). The cells sporadically showed immunoreactivity for S-100 protein and HMB-45 antibody (Figure 4, ×400). Extensive examination revealed no other skin, anal, facial, or rectal lesions. PET scan was performed and there was no metastatic lesion. On the basis of physical examination, histological and immunohistochemical studies, the diagnosis of PMME was made. 

## 3. Discussion

PMME is generally considered to be a highly malignant tumor which carries a poor prognosis and shows a rapidly fatal course [1]. The role of radiotherapy, chemotherapy, and immunotherapy is disappointing, and adjuvant treatment remains optional. In a series of 139 cases of PMME reviewed by Sabanathan et al. (67 treated by surgery alone; 72 treated with other modalities, such as chemotherapy and radiotherapy), the majority of these patients were diagnosed at advanced stages, and approximately 40% of patients had lymph node or distant metastases at the time of diagnosis. And only around 30% of the patients survived for more than 1 year after diagnosis in those 139 cases [3]. An accurate preoperative diagnosis of PMME is difficult to achieve due to lack of specificity of the symptoms and typical histological finding from endoscopic finding is abtainable only in less than 50%. Malignant melanoma, either esophageal primary or metastatic to esophagus, is difficult to distinguish from other esophageal malignancies clinically and histologically. It is often misinterpreted as negative or poorly differentiated squamous cell carcinoma when the melanoma cells contain either few or no melanin granules [5]. Etiology and natural course are not well known. It has been suggested that esophageal melanocytosis, a benign condition defined as an increased number of melanocyte in the basal layer with an increased quantity of melanin in these melanocytes, has been indicated as a premalignant lesion of PMME [6]. In 1970, Piccone et al. reported the first case of PMME with melanocytosis of the surrounding esophageal epithelium. A few cases of PMME with melanocytosis have been subsequently published, with melanocytosis being present in 25 percent of cases of PMME [7, 8]. Chronic stimuli such as the reflux of gastric juice and certain factors from adjacent neoplasm may also lead to melanocyte proliferation and the development of malignant melanoma [7]. 

Approximately 90% of the PMME are located in the distal two-thirds of the esophagus [4]. In majority of the cases, the malignant melanoma tended to be large, intraluminal, polypoid, and irregular surface [4, 5]. Microscopically, it usually involves the mucosal and submucosal layers, growing in a radial manner, and is composed of epithelioid cells arranged in nests or spindle cells arranged in fasicles with or without melanin pigment [9]. Eighty-five percent of PMME lesions are grossly pigmented, and 90% are pigmented microscopically. Esophagoscopy is helpful in demonstrating and localizing these lesions but definitive diagnosis is made by immunohistochemical staining with HMB-45 and S-100 on suspicious lesions with pigment or not [5]. In our case, the gross appearance demonstrated only flat and black pigmented mucosal lesions in distal esophagus with confirmation of histopathological finding. 

At the time of presentation of PMME, metastatic disease is present in about 50% of the patients, 31% hepatic, 29% mediastinal, 18% pulmonary, and 13% cerebral [9]. Sanchez AA et al. discovered some distinguishing points of PMME from metastatic melanoma by the presence of in situ melanoma, radial growth phase, melanocytosis and mixed epitheloid, and spindle cell morphology, in context of no history of melanoma [4]. Metastasis to esophagus seems to be a late event during the disease progression of cutaneous melanoma and is often associated with metastasis to other organs at the time of the diagnosis [4]. Complete history taking and physical examination are necessary in excluding the presence of a cutaneous or other primary tumor. A flurodeoxyglucose positron emission tomography (FDG-PET) scan has been used recently to detect metastatic lesions [7]. 

Extensive surgical resection, total or near-total esophagectomy with sufficient margin of resection, is first choice in treatment because of tendency to spread longitudinally along the submucosa [10]. Although the 5-year survival rate after a radical surgical resection has been reported to range from 10% to 48%, the prognosis for this disease remains dismal [7]. The role of radiotherapy, chemotherapy, and immunotherapy is disappointing, and adjuvant treatment remains optional [7]. Early detection, establishing a definitive diagnosis and effective treatment remain a challenge.

## References

[1] Li B, Lei W, Shao K (2007). Characteristics and prognosis of primary malignant melanoma of the esophagus. *Melanoma Research*.

[2] Vandewoude M, Cornelis A, Wyndaele D, Brussaard C, Kums R (2006). (18)FDG-PET-scan in staging of primary malignant melanoma of the oesophagus: a case report. *Acta Gastro-Enterologica Belgica*.

[3] Sabanathan S, Eng J, Pradhan GN (1989). Primary malignant melanoma of the esophagus. *The American Journal of Gastroenterology*.

[4] Sanchez AA, Wu T-T, Prieto VG, Rashid A, Hamilton SR, Wang H (2008). Comparison of primary and metastatic malignant melanoma of the esophagus: clinicopathologic review of 10 cases. *Archives of Pathology and Laboratory Medicine*.

[5] DeMatos P, Wolfe WG, Shea CR, Prieto VG, Seigler HF (1997). Primary malignant melanoma of the esophagus. *Journal of Surgical Oncology*.

[6] Chang F, Deere H (2006). Esophageal melanocytosis: morphologic features and review of the literature. *Archives of Pathology and Laboratory Medicine*.

[7] Oshiro T, Shimoji H, Matsuura F (2007). Primary malignant melanoma of the esophagus arising from a melanotic lesion: report of a case. *Surgery Today*.

[8] Suzuki H, Nakanishi Y, Taniguchi H (2008). Two cases of early-stage esophageal malignant melanoma with long-term survival. *Pathology International*.

[9] Kelly J, Leader M, Broe P (2007). Primary malignant melanoma of the oesophagus: a case report. *Journal of Medical Case Reports*.

[10] Volpin E, Sauvanet A, Couvelard A, Belghiti J (2002). Primary malignant melanoma of the esophagus: a case report and review of the literature. *Diseases of the Esophagus*.

